# The clinical outcomes of extended resections in patients with IV stage gallbladder cancers: A retrospective study from a large tertiary center

**DOI:** 10.3389/fonc.2022.1032737

**Published:** 2022-10-24

**Authors:** Kecheng Zhang, Hu Liu, Yongyang Zhao, Baohua Zhang

**Affiliations:** Department of Biliary Tract Surgery, Shanghai Eastern Hepatobiliary Surgery Hospital, Shanghai, China

**Keywords:** gallbladder carcinoma, extended resection, advanced stage, adverse events, long-term survival, recurrence

## Abstract

**Background and aim:**

The role of extended resections in patients with clinical stage IV gallbladder cancer (GBC) remains unclear. This study retrospectively analyzed the clinical outcomes of patients who underwent extended resections for IV GBC.

**Methods:**

Patients who were diagnosed with IV stage GBCs and underwent extended resections in Eastern Hepatobiliary Surgery Hospital, Shanghai, China, were retrospectively included in our study. Extended resection was defined as a major hepatectomy (resection of ≥3 liver segments), a pancreatoduodenectomy, or both. The clinical outcomes (baseline characteristics, preoperative variables, intraoperative variables, pathological outcomes, and follow-up data) were obtained and analyzed. The factors associated with major postoperative complications and long-term survival were analyzed by logistic regression analyses.

**Results:**

From January 2011 to June 2017, 74 patients were included in our study. There were 33 (44.6%) males and the median age was 62.5 years (interquartile range [IQR], 56.0-67.0 years). According to pathological specimens, the median tumor size was 7cm (IQR, 6-8cm), 73(98.6%) of them received R0 resection and 72 (97.2%) of them were IV A stage GBC. Three perioperative deaths (5.4%) occurred, and major postoperative complications occurred for 15 patients (20.3%). Among them, 61 patients (82.4%) experienced recurrence and 17 patients (23.0%) were still alive after a median follow-up period of 52 months. The disease free survival time was 9 months (95% confidence interval [CI], 7.8-10.2 months) and the overall survival was 18.0 months (95% CI, 15.2-20.8 months). Longer hospital stay days [odds ratio, (OR)=1.979, 95%CI:1.038-1.193, P=0.003), initial symptoms with abdominal pain (OR=21.489, 95%CI=1.22-37.57, P=0.036), more blood transfusion volume during hospitalization (OR=1.036, 95%CI:1.021-1.058, P=0.005), and intraoperative hemorrhage (OR=18.56, 95%C:3.54-47.65, P=0.001) were independently associated with postoperative complications. Moreover, locally recurrence (OR=1.65, 95%CI:1.17-1.96, P=0.015), and more adjuvant chemotherapy cycles (OR=1.46, 95%CI:1.13-1.76, P=0.026) were independently associated with long-term survival.

**Conclusion:**

Our retrospective study identified that extended resections can be safely and efficaciously performed on stage IV GBC patients in selected cases and performed by experienced surgeons.

## Introduction

Gallbladder cancer (GBC) is not common, but the malignancy nature of the cancer is highly aggressive. The incidence rate is less than 2/100,000 worldwide, with broad regional diversity (1, 2). The incidence of gallbladder cancer in China accounted for 0.4%~3.8% of all the biliary tract diseases, the disease 6th among digestive tract tumors (3, 4). Due to the lack of clinical symptoms in the early stages and the anatomical peculiarity of the absence of a submucosal layer, GBC is often diagnosed at an advanced stage (5). As a result, the prognosis for GBC is relatively poor, with the 5-year survival rate only reaching 5% in China (6).

The only curative treatment is complete surgical resection (7). However, only about one-tenth to one-fifth of patients are surgical candidates when diagnosed and they often underwent radical resections. But the prognosis of GBC after resection remains unfavorable (8, 9). Especially for patients with an advanced stage of GBC, the reported median overall survival (OS) period was only about 1 year after radical resection (10).

To achieve R0 resection, extended resections have been described in the treatment of advanced GBC. Comparing to radical resections, the extended resections have more extensive excision. These surgical methods include major hepatectomy, pancreatoduodenectomy (PD), and hepatectomy combined with PD (11–15). The survival benefits of extended resections for advanced GBC were inconsistent in these studies, but the reported post-operative adverse event rate is unexpectedly high (>50%). However, almost all published studies combined T3 and T4 GBC with a small sample size. The evidence level is not that high and we should not only follow the conclusions from these studies. More data is needed to show the actual clinical outcomes for stage IV GBC patients who underwent extended resections.

The objective of this study is to analyze the results of extended resections for patients with stage IV stage GBC with a study cohort in a large volume center. The primary aim is post-operative survival. The second aim is to explore the factors associated with long-term survival and postoperative major complications.

## Materials and methods

### Study cohort

**Figure 1:**
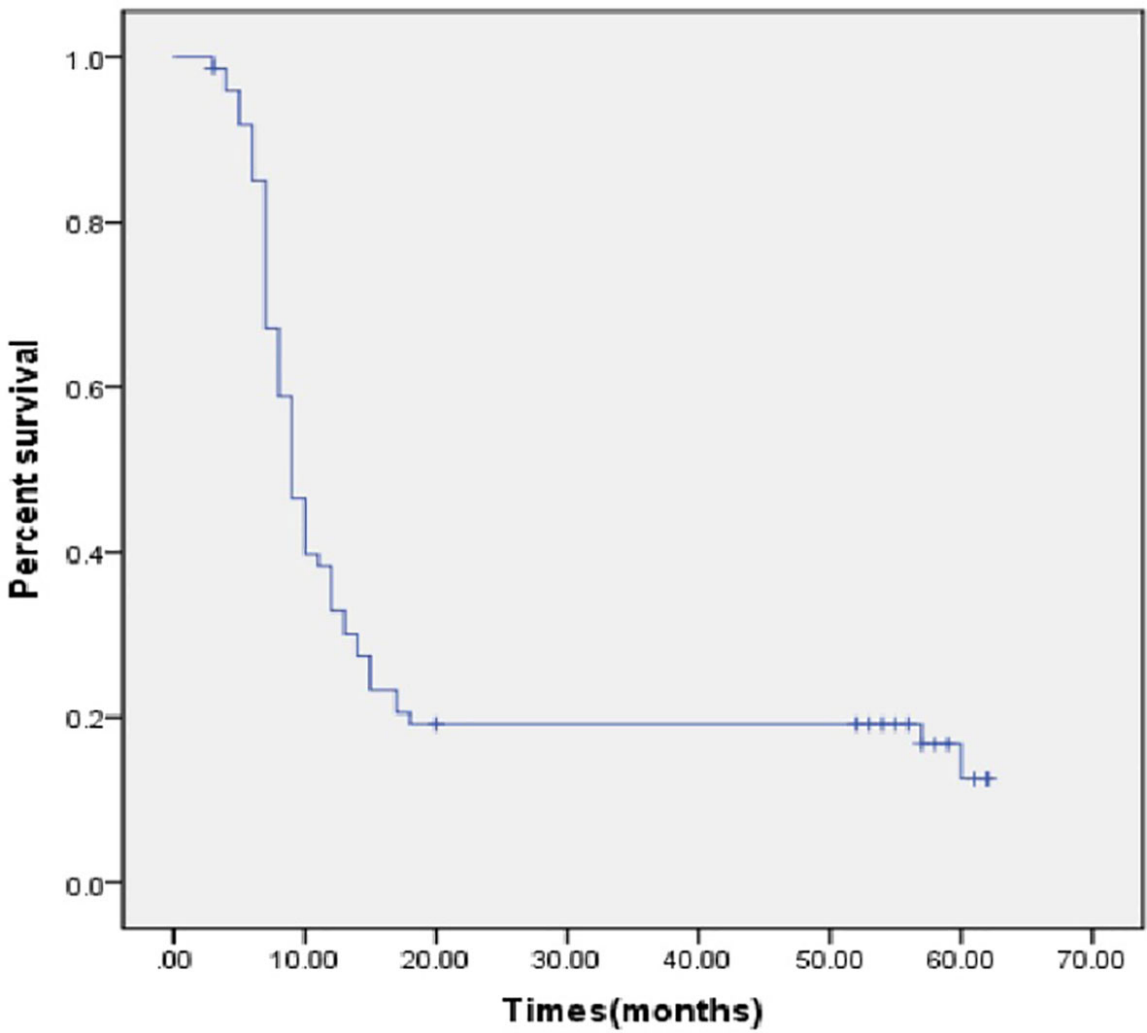
Disease free survival in months.

From January 2011 to June 2017, patients who were diagnosed with IV A stage GBCs and underwent extended resections in the department of biliary surgery, Eastern Hepatobiliary Surgery Hospital, Shanghai, China, were retrospectively included in our study. The following inclusion criteria were applied: (1) patients pathologically diagnosed with GBC; (2) patients whose full electronic medical records and imaging records could be obtained; and (3) patients whose follow-up data could be obtained. Exclusion criteria included (1) specimens obtained from reresections; (2) concomitant other neoplasms on final pathology (e.g., primary liver cancer, cholangiocarcinoma); and (3) patients with unavailable pathological and follow-up data.(4) patients were incidentally diagnosed with GBC. The selection procedure of the study participants is presented in **Figure 1**. The Institutional Review Board of our hospital approved the study protocol.

The grade of T4 stage was according to the American Joint Committee on Cancer (AJCC) 8th edition. In our study, if a patient was staged with IV A, he/she had T4 (invasion of the portal vein or main hepatic artery, or direct invasion of two or more extrahepatic organs or structures), N0-1 (one to three regional lymph nodes had metastases) and M0. If a patient was staged with IV B, he/she had any T, N2 (more than four regional lymph nodes had metastases), and M0 (patients with M1 will not receive surgical resection).

The decision on surgical treatment was made by a multidisciplinary hepatopancreatobiliary team. The choices of surgical procedures depended on the location and extent of diseases obtained by imaging modalities. All the operations were performed by experienced surgeons. Long-term survival was defined as survival for 2 years or longer.

### Perioperative management and chemotherapy strategy

Routine preoperative exams were carried out before surgery to rule out any surgical contraindications. The electrocardiograph, lung function, coagulation function, liver function, renal function, electrolyte, and hemoglobin were among the standard tests performed. One hour before the surgery, antibiotic prophylaxis (third generation cephalosporin or ciprofloxacin) was routinely used. After preoperative evaluations, all the patients were supposed to have good surgical tolerance (American Society of Anesthesiologists grade II or above). Informed consent was provided by every patient. All operations were performed by experienced surgeons.

After surgery, antibiotics (third generation cephalosporin) were routinely used and lasted for a week. Blood examinations were performed to determine whether infections and electrolyte disturbances existed. Total parenteral nutrition was performed before the recovery of intestinal function. Plain CT was performed if any clinically significant symptoms occurred to exclude postoperative complications.

Adjuvant chemotherapy will be recommended for patients after surgical resection if they can tolerate it. Most of the regimens used for post-operative adjuvant chemotherapy were oxaliplatin+ S-1/capecitabine. Post-operative adjuvant radiation was not routinely performed in our hospital.

If a patient experiences a recurrence, chemotherapy with palliative intent will be recommended. The most frequently used regimen was S-1/capecitabine.

### Variable definitions

Various indicators were included in our study to fully illustrate the impact of extended resections on patients with stage IV GBC.

First, the baseline characteristics, including age, sex, body mass index (BMI), initial symptoms, hospital stay days, and surgical methods. Major hepatectomy (resection of three or more segments of the liver), PD, or both, with or without en bloc resection of the duodenum, colon, or stomach, were the surgical methods used in our study (14). Enlarged lymph node dissection and biliary anastomosis were performed in each case.

Second, the preoperative variables, including total bilirubin, alanine aminotransferase (ALT), carcinoembryonic antigen (CEA), carbohydrate antigen 19-9 (CA19-9) and the history of biliary drainage [through Endoscopic Retrograde Cholangiopancreatography (ERCP) or percutaneous transhepaticcholangial drainage (PTCD)].

Third, the intraoperative variables, including operation duration, intraoperative blood loss, blood transfusion volume, and intraoperative complications.

Fourth, the pathological outcomes included tumor size, location (gallbladder neck, body and bottom), differentiated degree, margin status, and peripheral tissue invasion status. Peripheral tissue invasion status consisted of perineural invasion, vascular invasion, cancerization of ducts, lymphatic metastasis, and common bile duct invasion.

Fifth, the follow-up data, including perioperative deaths(within 30 days after surgery), post- operative major complications, post-operative adjuvant chemotherapy history, disease-free survival (DFS) rate, and overall survival (OS) rate. The number of months between the date of extended resection or the date of the final follow-up examination was used to determine DFS. Recurrence was defined as a local or metastatic tumor confirmed by radiology or histology during postoperative follow-up. The OS calculation took into account all types of fatalities. Most of the regimens used for post-operative adjuvant chemotherapy were oxaliplatin+ S-1/capecitabine. Post-operative adjuvant radiation was not routinely performed in our hospital. Major complications were defined as Clavien-Dindo grade III or higher (16).

### Statistical analysis

Continuous variables are presented as median (interquartile range), and categorical data are presented as number (%). Survival was reported using Kaplan-Meier methods. The differences between groups were compared by student’s t-test, Mann-Whitney U test, chi-square test, and Fisher’s exact test. Logistic regression analysis was used to identify factors associated with long-term survival and decreased adverse events. Variables of potential significance (P < 0.10) were entered into the Logistic regression model. All models were adjusted for age and sex, and the results were presented as an odds ratio (OR) with a 95% confidence interval (CI). All p values of 0.05 or lower were considered statistically significant. All statistical analyses were performed using SPSS version 22.0 (IBM Corporation, Armonk, NY, USA).

## Results

### Baseline characteristics

Between January 2011 and June 2017, 345 patients underwent extended resection with curative intent in our hospital. After inclusion and exclusion criteria were applied to the study cohort, 74 stage IV GBC patients were finally included in our study.

Among the 74 patients, there were 33 (44.6%) males and 41 (55.4%) females. At the time of diagnosis, the median age was 62.5 years (interquartile range [IQR], 56.0-67.0 years). The median BMI was 23.27 (IQR, 21.47-24.90) with 28 patients(37.8%) having a BMI over 24. The median hospital stay day was 23 days (IQR,18-31 days). The initial presenting symptoms were jaundice (n = 38), abdominal pain (n = 49), nausea (n = 2), weight loss (n = 1), back pain (n = 1), and no symptoms (n=1). 18 (24.3%) of them had multiple presenting symptoms. The median hospital stay day was 23 days (IQR, 18-31 days). 56 patients (75.7%) underwent major hepatectomy, 1 patient (1.4%) underwent PD, and 17 patients (23.0%) underwent major hepatectomy combined with PD (**Table 1**).

**Table 1:** The baseline characteristics and perioperative variables of gallbladder cancer patients who underwent extended resection.

Variables	Total (n=74)	%
Age, median(IQR)	62.5	56.0-67.0
Gander, male	33	44.6
BMI, median(IQR)	23.27	21.47-24.90
>24	28	37.8
Hospital stays, median days(IQR)	23	18-31
Initial presenting symptoms		
jaundice	38	51.4
abdominal pain	49	66.2
nausea	2	2.7
weight loss	1	1.4
back pain	1	1.4
no symptom	1	1.4
Type of surgery		
major hepatectomy	56	75.7
PD	1	1.4
major hepatectomy+PD	17	23.0
CA19-9 level, median, ku/L(IQR)	287	64.8- 880.8
>37 ku/L	65	87.8
CEA level, median, ng/mL(IQR)	3.35	2.30 - 5.45
>5 ng/mL	22	29.7
Total bilirubin levels, median, umol/L(IQR)	55.25	11.25 - 202.5
>18 umol/L	47	63.5
ALT, median, U/L(IQR)	69	20.75 - 162
>40 U/L	50	67.6
Preoperative biliary drainage		
PTCD	25	33.8
ERCP	7	9.5
Operation duration, median min(IQR)	330	292.5-412.5
Intraoperatve blood loss, median ml (IQR)	400	250-725
Blood transfusion volume, median ml (IQR)	800	0-1600
Intraoperative hemorrhage	4	5.4

IQR, interquartile range; PD, pancreatoduodenectomy; BMI, body mass index; CEA, carcinoembryonic antigen; ALT, alaninetransaminase; PTCD, percutaneous transhepaticcholangial drainage; ERCP, Endoscopic Retrograde Cholangiopancreatography.

### Perioperative variables

At presentation, the median value of CA19-9 was 287 ku/L(IQR 64.8- 880.8 ku/L), and 65 (87.8%) of them had elevated CA19-9 value (>37 ku/L). The median value of CEA was 3.35 ng/mL(IQR 2.30 - 5.45 ng/mL), and only 22 (29.7%) of them had elevated CEA value (>5 ng/mL). At presentation, the median total bilirubin level was 55.25 umol/l (IQR, 11.25 - 202.5 umol/L), and 47 (63.5%) of them had elevated total bilirubin levels(>18 umol/L). The median value of ALT was 69 U/L(IQR 20.75 - 162 U/L), and 50 (67.6%) of them had elevated ALT value (>40 U/L). 32 (43.2%) of them underwent preoperative biliary drainage (25 patients through PTCD and 7 patients through ERCP)(**Table 1**).

For intraoperative variables, the median operation duration was 330 mins (IQR, 292.5-412.5 mins) including operation duration, the median intraoperatve blood loss was 400ml (IQR, 250ml-725ml), and the median blood transfusion volume during hospitalization was 800ml (IQR, 0-1600ml). Intraoperative complications occurred for 4 patients (5.4%), and all the complications were hemorrhagic (**Table 1**).

### Pathological outcomes

According to pathological specimens, the median tumor size was 7 cm (IQR, 6-8 cm). 12 (16.2%), 13 (17.6%) and 28 (37.8%) tumors originated from the bottom, body, and neck of the gallbladder. The location of tumors could not be identified in 21 (28.4%) patients due to the deep invasiveness of the tumors. Most of them were moderately differentiated (n = 67, 90.5%), 5 (6.8%) were identified as being poorly differentiated, and 2 (2.7%) were identified as being well differentiated.

After resection, only 1 patient (1.4%) received R1 resection due to the margin status being positive. The other 73 patients were identified to have received R0 resection and their margin status was all negative. But, contrary to the good results of the margin status, 36 patients (48.6%) had perineural invasion, 59 patients (79.7%) had vascular invasion, 11 patients (14.9%) had invasion of the common bile duct, and only 4 patients (5.4%) had cancerization of the duct. According to pathological specimens, 25 patients (33.8%) had N1 lymphatic metastases, 2 patients (2.7%) had N2 lymphatic metastases, and the other patients had N0 lymphatic metastases. Finally, 72 patients were staged as IV A GBC and 2 patients were staged as IV B GBC. Histology showed adenocarcinoma (n = 67), squamous-cell carcinoma (n = 4), adenosquamous carcinoma (n = 2), and neuroendocrine tumor (n = 1) **(** **Table 2** **)**.

**Table 2:** The pathological outcomes and long-term follow up data of gallbladder cancer patients who underwent extended resection.

Variables	Total (n=74)	%
Tumor size, median cm (IQR)	7	6-8
Tumor location		
Bottom	12	16.2
Body	13	17.6
Neck	28	37.8
Uncertain	21	28.4
Tumor differentiation		
Well	5	6.8
Moderately	67	90.5
Poorly	2	2.7
Margin status		
negative	73	98.6
positive	1	1.4
Peripheral tissue invasion status		
perineural invasion	36	48.6
vascular invasion	59	79.7
common bile duct invasion	11	14.9
cancerization of duct	4	5.4
Lymphatic metastasis status		
N0	47	63.5
N1	25	33.8
N2	2	2.7
GBC staging		
IV A	72	97.3
IV B	2	2.7
Types of histology		
adenocarcinoma	67	90.5
squamous-cell carcinoma	4	5.5
adenosquamous carcinoma	2	2.7
neuroendocrine tumor	1	1.3
Major postoperative complications	15	20.3
infection	9	12.2
delayed gastric emptying	4	5.5
biliary fistula	1	1.3
others	4	5.5
Recurrence sites	61	82.4
locally	36	48.6
distant	25	33.8
Adjuvant chemotherapy cycles, median n (IQR)	6.0	3.0-9.0

IQR, interquartile range; GBC, Gallbladder cancer.

### Long-term follow-up data

Three perioperative deaths (5.4%) occurred, two due to sepsis and one due to liver failure. Major postoperative complications occurred for 15 patients (20.3%), including infection(n = 9), delayed gastric emptying (n = 4), biliary fistula (n = 1), and others (n = 4). The median number of adjuvant chemotherapy cycles was 6.0 (IQR 3.0–9.0).

Recurrence occurred for 61 patients (82.4%) after a median disease-free interval of 9 months (95% confidence interval [CI], 7.8–10.2 months, **Figure 1**). Imaging data showed that there were 36 cases that had local recurrence and 25 cases had distant recurrence (on the peritoneum, n = 12, in the lung, n = 10, other locations, n = 3).

**Figure 2:**
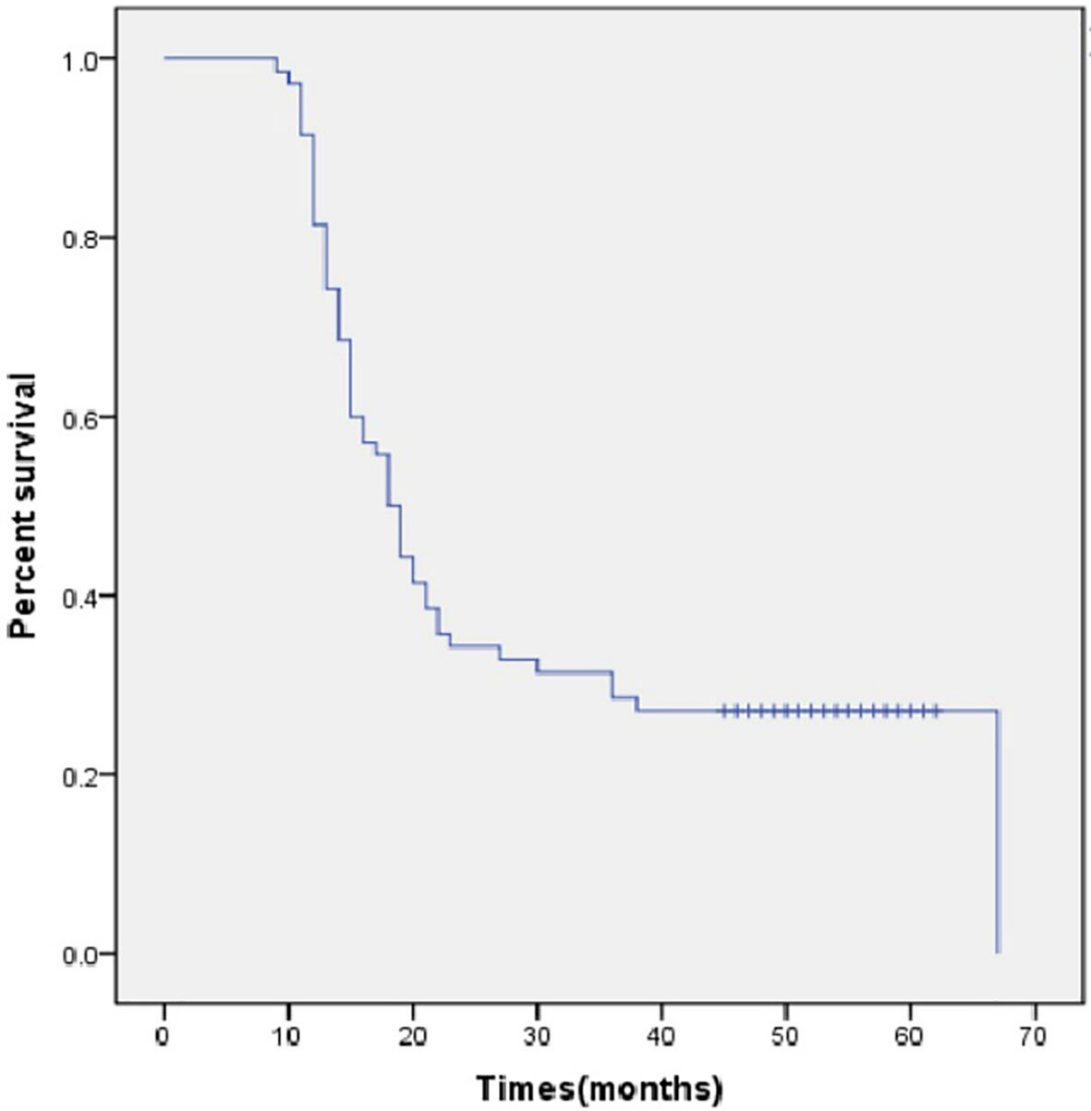
Overall survival in months.

All the deaths were tumor-related. After a median follow-up period of 52 months, 26 patients (35.1%) survived beyond 2 years, and 17 patients (23.0%) were still alive at the time of last follow-up. The median OS was 18.0 months (95% CI, 15.2-20.8 months, **Figure 2**) and the median OS was 18.0 months (95% CI, 16.0–20.0 months) when the perioperative deaths were excluded.

#### Factors associated with postoperative complications in patients with GBC who underwent extended resections

A logistic regression model was established to identify factors associated with postoperative complications. The baseline characteristics, perioperative variables, and intraoperative variables were included in the univariate analysis. The differences in variables between the patients with or without postoperative complications were compared and presented in **Supplementary Table 1** (without age and gender adjusted) and **Table 3** (with age and gender adjusted**)**, and the patients who experienced perioperative death were excluded (n = 3). Finally, a total of 14 GBC patients (14/71, 19.7%) experienced postoperative complications after extended resections.

**Table 3:** Univariate and multivariable analyses of risk factors for postoperative complications.

Parameters	With complications (n=14)	Without complications (n=57)	p value for univariate analyses	Adjusted OR (95% CI)	p value for multivariate analyses
**Age**,mean ± SD,year	63.4 ± 7.1	62.0 ± 8.4	0.578	1.640(0.336-7.999)	0.540
**Male, n%**	6(42.6)	26(45.6)	0.853	1.058(0.951-1.177)	0.299
**Hospital stay days**, mean ± SD	38.3 ± 14.8	24.1 ± 9.6	0.013	1.979(1.038-1.193)	0.003
**BMI, mean ± SD**	23.3 ± 3.2	23.3 ± 2.7	0.939		
**Initial presenting symptoms, n%**					
jaundice	5(35.7)	31(54.4)	0.211		
abdominal pain	13(92.9)	35(61.4)	0.024	21.489(1.22-37.57)	0.036
**Type of surgery, n%**					
major hepatectomy	10(71.4)	45(78.9)	0.546		
major hepatectomy+PD	4(28.6)	12(21.1)			
**CA19-9 level,** mean ± SD	408.7 ± 432.1	585.4 ± 710.8	0.378		
**CEA level,** mean ± SD	11.3 ± 24.8	7.0 ± 9.6	0.538		
**Total bilirubin levels,** mean ± SD	129.8 ± 149.5	107.4 ± 121.8	0.558		
**ALT levels,** mean ± SD	137.8 ± 163.1	104.5 ± 106.4	0.351		
**Preoperative biliary drainage, n%**	6(42.9)	25(43.9)	0.865		
**Operation duration,** mean ± SD	375 ± 38.7	329.8 ± 79.2	0.280		
**Intraoperatve blood soss,** mean ± SD	614.3 ± 293.1	460.5 ± 319.8	0.099	1.001(0.998-1.004)	0.514
**Blood transfusion volume,** mean ± SD	2332.1 ± 2773.2	1126.3 ± 2689.7	0.016	1.036(1.021-1.058)	0.005
**Intraoperative hemorrhage,n%**	4(28.6)	0(0)	0.001	18.56(3.54-47.65)	0.001
**Tumor size,** mean ± SD	7.05 ± 1.37	7.53 ± 2.68	0.371		
**Tumor location**					
Bottom	7(12.3)	3(21.4)	0.378		
Body	10(17.5)	3(21.4)	0.736		
.Neck	23(40.4)	5(35.7)	0.750		
Uncertain	17(29.8)	3(21.4)	0.531		

IQR, interquartile range; PD, pancreatoduodenectomy; BMI, body mass index; CEA, carcinoembryonic antigen; ALT, alaninetransaminase.

Based on univariate analysis, longer hospital stay days (*P*=0.013), initial symptoms with abdominal pain (*P*=0.024), more intraoperatve blood loss (*P*=0.099), more blood transfusion volume during hospitalization (*P*=0.016) and intraoperative hemorrhage (*P*=0.001) were closely correlated with postoperative complications. These factors were included in the multivariate logistic regression analysis (**Table 3**).

Based on multivariate logistic regression analysis, longer hospital stay days (OR=1.979, 95%CI:1.038-1.193, *P*=0.003), initial symptoms with abdominal pain (OR=21.489, 95%CI=1.22-37.57, *P*=0.036), more blood transfusion volume during hospitalization (OR=1.036, 95%CI:1.021-1.058, *P*=0.005), and intraoperative hemorrhage (OR=18.56, 95%C:3.54-47.65, *P*=0.001) were independently associated with postoperative complications after age and gender were adjusted (**Table 3**).

#### Factors associated with long-term survival in patients with GBC who underwent extended resections

Patients who survived longer than 2 years were defined as long-term survivors in our study. All the variables were included in the univariate analysis. The differences in variables between the patients with or without postoperative complications were compared and presented in **Supplementary Table 2** (without age and gender adjusted) and **Table 4** (with age and gender adjusted). The analysis of margin status was excluded because only one patient had positive margin status.

**Table 4:** Univariate and multivariable analyses of risk factors for long-term survival.

Parameters	Long-term survival (n=26)	Short-term survival (n=48)	p value for univariate analyses	Adjusted OR (95% CI)	p value for multivariate analyses
**Age**,mean ± SD,year	60.7 ± 7.7	63.0 ± 8.1	0.246	0.626(0.228-1.722)	0.364
**Male,** n%	13(50.0)	20(41.7)	0.491	0.967(0.908-1.030)	0.297
**Hospital stay days**, mean ± SD	26.3 ± 13.5	27.4 ± 11.9	0.726		
**BMI,** mean ± SD	23.4 ± 2.6	23.2 ± 2.8	0.705		
**Initial presenting symptoms,** n%					
jaundice	12(46.2)	26(54.2)	0.510		
abdominal pain	17(65.4)	32(66.7)	0.911		
**Type of surgery,** n% (n=73)					
major hepatectomy	21(80.8)	35(74.5)*	0.542		
major hepatectomy+PD	5(19.2)	12(25.5)			
**CA19-9 level,** mean ± SD	517.8 ± 667.0	594.5 ± 679.3	0.640		
**CEA level,** mean ± SD	5.7 ± 8.3	8.9 ± 15.7	0.341		
**Total bilirubin levels,** mean ± SD	109.9 ± 118.8	117.1 ± 133.1	0.819		
**ALT levels,** mean ± SD	144.9 ± 159.8	92.1 ± 82.5	0.125		
**Preoperative biliary drainage,** n%	10(38.5)	22(45.8)	0.541		
**Operation duration,** mean ± SD	337.8 ± 80.3	340.3 ± 73.9	0.936		
**Intraoperatve blood soss,** mean ± SD	530.8 ± 342.7	488.5 ± 319.6	0.591		
**Blood transfusion volume,** mean ± SD	1409.6 ± 1867.2	1459.4 ± 3091.3	0.941		
**Intraoperative hemorrhage,**n%	0(0)	4(8.3)	0.291		
**Tumor size,** mean ± SD	7.02 ± 1.97	7.49 ± 2.84	0.461		
**Tumor location**					
Bottom	3(11.5)	9(18.8)	0.354		
Body	4(15.4)	9(18.8)	0.618		
Neck	13(50)	15(31.3)	0.112		
Uncertain	6(23.1)	15(31.3)	0.356		
**Tumor differentiation**					
Well	1(3.8)	4(8.3)	0.651		
Moderately	24(92.3)	43(89.6)	0.702		
Poorly	1(3.8)	1(2.1)	1.00		
**Peripheral tissue invasion status**					
perineural invasion	11(42.3)	25(52.1)	0.422		
vascular invasion	21(80.8)	38(79.2)	0.931		
common bile duct invasion	6(23.1)	5(10.4)	0.144		
cancerization of duct	1(3.8)	3(6.3)	0.756		
**Lymphatic metastasis status**					
N0	17(65.4)	30(62.5)	0.806		
N1	7(26.9)	18(37.5)	0.358		
N2	2(7.7)	0	0.12		
**GBC staging**					
IV A	24(92.3)	48(100)	0.12		
IV B	2(7.7)	0(0)			
**Types of histology**					
adenocarcinoma	22(84.6)	45(93.8)	**0.048**	0.32(0.10-1.691)	0.180
squamous-cell carcinoma	2(7.7)	2(4.2)	0.522		
adenosquamous carcinoma	1(3.8)	1(2.1)	0.655		
neuroendocrine tumor	1(3.8)	0(0)	0.351		
**Major postoperative complications**	15	20.3			
infection	2(7.7)	4(8.3)	0.923		
delayed gastric emptying	1(3.8)	3(6.3)	0.662		
biliary fistula	0(0)	1(2.1)	1.00		
others	1(3.8)	3(6.3)	0.662		
**Recurrence sites** (n=61)					
locally	16(76.2)	17(42.5)	**0.012**	1.65(1.17-1.96)	0.015
distant	5(23.8)	23(57.5)			
**Adjuvant chemotherapy cycles,** mean ± SD	8.20 ± 2.52	4.50 ± 3.50	**0.016**	1.46(1.13-1.76)	0.026

SD, Standard deviation; GBC, Gallbladder cancer; IQR, interquartile range; PD, pancreatoduodenectomy; BMI, body mass index; CEA, carcinoembryonic antigen; ALT, alaninetransaminase. Bold values: P<0.1 and were included in multivariate analyses.* only one case underwent PD in short-term survival group and was not included in analysis.

Based on univariate analysis, histology types of adenocarcinoma (*P*=0.048), locally recurrence (*P*=0.012), and more adjuvant chemotherapy cycles (*P*=0.016) were closely correlated with long-term survival. These factors were included in the multivariate logistic regression analysis (**Table 4**).

Based on multivariate logistic regression analysis, locally recurrence (OR=1.65, 95%CI:1.17-1.96, *P*=0.015), and more adjuvant chemotherapy cycles (OR=1.46, 95%CI:1.13-1.76, *P*=0.026) were independently associated with long-term survival after age and gender were adjusted (**Table 4**).

## Discussion

This retrospective study investigated the clinical outcomes of extended resections in patients with stage IV GBC. To date, this study has the largest sample size on this issue. Our study cohort identified that extended resections can be safely and efficaciously performed on stage IV GBC patients in large volume centers. Despite a reported median disease-free survival of 9 months and a median OS of 18.0 months, 35% of the patients survived beyond 2 years, and 23% were still alive after a median follow-up time of 52 months. About one-fifth of patients experienced major postoperative complications, and the perioperative deaths were less than 5%. Moreover, we identified that longer hospital stay days, initial symptoms with abdominal pain, more blood transfusion volume during hospitalization, and intraoperative hemorrhage were independently associated with major postoperative complications and local recurrence, and more adjuvant chemotherapy cycles were associated with long-term survival.

The value of extended resections for advanced GBC remains controversial. Results from previous studies were inconsistent. Two Japanese retrospective studies showed no survival benefit for advanced bile duct cancer and GBC patients who underwent hepatopancreaticoduodenectomy. No patient survived beyond 2 years, and the R0 resection rate was only 20% (17, 18). In a study from the Memorial Sloan Kettering Cancer Center (11), perioperative mortality rate was14% (5 of 36 patients) for major hepatectomies. Recurrence occurred for 24 patients (73%) and R0 resection margins were achieved for 91% of the patients. Most importantly, the 5-year survival rate was 27%. The R0 resection rate and 5-year survival rate were comparable with our study. They concluded that major hepatectomy combined with PD is appropriate in certain cases. However, the surgical indications were still not clear. Another study by Fong et al. (19) concluded that radical resection can provide long-term survival, even for large tumors with extensive liver invasion. However, the multi-variable analysis in that study showed that the extent of liver resection was not a risk factor for long-term survival. Due to the emerging evidence from previous studies, we conducted this study to explore the impact of extended resections for stage IV GBC. We believe our study can significantly help establish the role of extended resections in the management of stage IV GBC.

Searching the prognosis data about the advanced GBC patients who did not receive surgical resections, a Netherland population-based study that the 1-year survival rate for patients with advanced unresected GBC is less than 10% (20). The median OS of all patients who had unresected GBC treated with palliative chemotherapy was 6.4 months (14). Moreover, in a large scale randomized controlled ABC-02 trial (gemcitabine + cisplatin vs gemcitabine alone for unresected biliary tract cancer), no patient survived beyond 3 years (21). In our study, the 2 year survival rate was 35.1%, the estimated 5-year survival rate was about 20% and median OS was 18 months. The prognosis of advanced GBC patients was significantly improved by extended resection. However, more prospective randomized controlled studies are needed to further confirm the conclusion.

Almost all the patients reached R0 resection in our study. The R0 resection was higher than in previous studies. The first reason may be due to the careful patient selection preoperatively. The contrast-enhanced CT and MRI images will be carefully reviewed by radiologists and surgeons. Large tumors with extensive liver invasion will not perform extended resections. Second, the aim of the surgery is R0 resections. If the surgeon found there was no possibility to reach R0 resection during operation. The surgery will not continue and only a biopsy will be performed. Third, the surgeons who were qualified to perform extended resection were all rich in experience. On average, they perform more than 200 biliary and pancreatic surgeries every year. Therefore, most of them were IV A stage GBC and a high R0 resection rate was achieved in our study cohort.

Reducing the major postoperative complications is a way to improve prognosis. We identified that longer hospital stay days, more blood transfusion volume during hospitalization, and intraoperative hemorrhage were independently associated with major postoperative complications. These factors were interrelated. More blood transfusions are required when intraoperative hemorrhage occurs. The complications due to hypovolemia require more hospital days for recovery. Therefore, more detailed intraoperative procedures are required to prevent intraoperative hemorrhage and finally reduce the major postoperative complications. Moreover, the initial symptoms of abdominal pain were also identified as a risk factor for major postoperative complications. The abdominal pain caused by extensive nerve invasion requires a larger resection area and finally causes intraoperative hemorrhage. However, the assumption should be verified in further studies.

For effective patient selection, prognostic factors for long-term survival should be identified. In our study, no pathological outcomes were associated with poor prognosis. However, poor survival in other studies was associated with preoperative tumor biomarker level, perivascular invasion, and invasion of the liver parenchyma (18, 22–24). All clinical stages of GBC were included in these studies with the small sample size. The IV stage GBCs were already aggressive tumors; therefore, these factors may not influence prognosis. Adjuvant chemotherapy is acknowledged as the first line treatment after surgical resection in most cancers. The recent BILCAP trial concluded that adjuvant capecitabine can improve OS for patients with resected biliary tract cancer (25). Another recent study found that individuals with resected GBC who had adjuvant chemoradiation treatment had considerably lower recurrence rates and improved OS than those that underwent surgery alone. Notably, individuals with N0 disease did not appear to benefit from adjuvant therapy. Therefore, this effect was only shown in patients with positive lymph node status (26).However, the role of adjuvant chemotherapy in advanced GBC after surgical resection is still not clear. Our study showed more adjuvant chemotherapy cycles were associated with long-term survival. However, no comparison group was set in our study. Further prospective randomized control is needed to clarify the true value of adjuvant chemotherapy and the optimal regimens.

Our study had several limitations. First, it was a retrospective review of a cohort of patients in a single center, so some selection bias may exist. The small sample size made it impossible to draw statistical conclusions about prognostic factors for prolonged survival, especially the pathological outcomes. Second, due to the retrospective nature, the postoperative quality of life was not analyzed. Third, the high morbidity and mortality rates associated with extensive resections should be weighed against the apparent survival benefit. Therefore, further prospective randomized control with a larger cohort of patients is needed to clarify the true value of extended resections.

## Conclusion

Our retrospective study identified that extended resections can be safely and efficaciously performed on stage IV GBC patients in large volume centers. The median disease-free survival was 9 months and the median OS was 18.0 months, 35% of the patients survived beyond 2 years, and 23% were still alive after a median follow-up time of 52 months. About one-fifth of patients experienced major postoperative complications, and the perioperative deaths were less than 5%. Longer hospital stay days, initial symptoms with abdominal pain, more blood transfusion volume during hospitalization, and intraoperative hemorrhage were independently associated with major postoperative complication and locally recurrence and more adjuvant chemotherapy cycles were associated with long-term survival. Therefore, extended resections for patients with IVA stage GBC should be considered in selected patients and performed by experienced surgeons.

## Data availability statement

The raw data supporting the conclusions of this article will be made available by the authors, without undue reservation.

## Ethics statement

Written informed consent was obtained from the individual(s), and minor(s)’ legal guardian/next of kin, for the publication of any potentially identifiable images or data included in this article.

## Author contributions

KZ: Data collection, statistical analysis and interpretation of data. HL: Paper writing. BZ: Design of the study, data collection and literature search. YZ: Manuscript review design of the study. All authors contributed to the article and approved the submitted version.

## Funding

This study was supported by Mengchao talents foundation from Eastern Hepatobiliary Surgery Hospital.

## Conflict of interest

The authors declare that the research was conducted in the absence of any commercial or financial relationships that could be construed as a potential conflict of interest.

## Publisher’s note

All claims expressed in this article are solely those of the authors and do not necessarily represent those of their affiliated organizations, or those of the publisher, the editors and the reviewers. Any product that may be evaluated in this article, or claim that may be made by its manufacturer, is not guaranteed or endorsed by the publisher.
