# Supplementary material for: The clinical outcomes of extended resections in patients with IV stage gallbladder cancers: A retrospective study from a large tertiary center
Source: Front Oncol. 2022 Oct 24;12:1032737. doi: 10.3389/fonc.2022.1032737 (PMC9638100; doi:10.3389/fonc.2022.1032737)
Supplement: Supplementary file 1 [file Table_1.docx]

**Supplementary table 1.** Univariate and multivariable analyses of risk factors for postoperative complications before age and gender adjusted.

| Parameters | With complications (n=14) | Without complications (n=57) | p value for univariate analyses | OR (95% CI) | p value for multivariate analyses |
| --- | --- | --- | --- | --- | --- |
| **Age**,mean±SD ,year | 63.4±7.1 | 62.0±8.4 | 0.578 |  |  |
| **Male, n%** | 6(42.6) | 26(45.6) | 0.853 |  |  |
| **Hospital stay days**, mean±SD | 38.3±14.8 | 24.1±9.6 | 0.013 | 1.932(1.037-1.190) | 0.005 |
| **BMI, mean±SD** | 23.3±3.2 | 23.3±2.7 | 0.939 |  |  |
| **Initial presenting symptoms, n%** |  |  |  |  |  |
| jaundice | 5(35.7) | 31(54.4) | 0.211 |  |  |
| abdominal pain | 13(92.9) | 35(61.4) | 0.024 | 20.654(1.21-36.89) | 0.039 |
| **Type of surgery, n%** |  |  |  |  |  |
| major hepatectomy | 10(71.4) | 45(78.9) | 0.546 |  |  |
| major hepatectomy+PD | 4(28.6) | 12(21.1) |  |  |  |
| **CA19-9 level,** mean±SD | 408.7±432.1 | 585.4±710.8 | 0.378 |  |  |
| **CEA level,** mean±SD | 11.3±24.8 | 7.0±9.6 | 0.538 |  |  |
| **Total bilirubin levels,** mean±SD | 129.8±149.5 | 107.4±121.8 | 0.558 |  |  |
| **ALT levels,** mean±SD | 137.8±163.1 | 104.5±106.4 | 0.351 |  |  |
| **Preoperative biliary drainage, n%** | 6(42.9) | 25(43.9) | 0.865 |  |  |
| **Operation duration,** mean±SD | 375±38.7 | 329.8±79.2 | 0.280 |  |  |
| **Intraoperatve blood soss,** mean±SD | 614.3±293.1 | 460.5±319.8 | 0.099 | 1.001(0.997-1.005) | 0.516 |
| **Blood transfusion volume,** mean±SD | 2332.1±2773.2 | 1126.3±2689.7 | 0.016 | 1.034(1.019-1.056) | 0.005 |
| **Intraoperative hemorrhage,n%** | 4(28.6) | 0(0) | 0.001 | 17.36(3.34-46.03) | 0.001 |
| **Tumor size,** mean±SD | 7.05±1.37 | 7.53±2.68 | 0.371 |  |  |
| **Tumor location** |  |  |  |  |  |
| Bottom | 7(12.3) | 3(21.4) | 0.378 |  |  |
| Body | 10(17.5) | 3(21.4) | 0.736 |  |  |
| .Neck | 23(40.4) | 5(35.7) | 0.750 |  |  |
| Uncertain | 17(29.8) | 3(21.4) | 0.531 |  |  |

IQR: interquartile range; PD: pancreatoduodenectomy; BMI: body mass index; CEA: carcinoembryonic antigen; ALT: alaninetransaminase;
