# Supplementary material for: The clinical outcomes of extended resections in patients with IV stage gallbladder cancers: A retrospective study from a large tertiary center
Source: Front Oncol. 2022 Oct 24;12:1032737. doi: 10.3389/fonc.2022.1032737 (PMC9638100; doi:10.3389/fonc.2022.1032737)
Supplement: Supplementary file 2 [file Table_2.docx]

**Supplementary table 2.** Univariate and multivariable analyses of risk factors for long-term survival before age and gender adjusted.

| Parameters | Long-term survival (n=26) | Short-term survival (n=48) | p value for univariate analyses | Adjusted OR (95% CI) | p value for multivariate analyses |
| --- | --- | --- | --- | --- | --- |
| **Age**,mean±SD ,year | 60.7±7.7 | 63.0±8.1 | 0.246 |  |  |
| **Male,** n% | 13(50.0) | 20(41.7) | 0.491 |  |  |
| **Hospital stay days**, mean±SD | 26.3±13.5 | 27.4±11.9 | 0.726 |  |  |
| **BMI,** mean±SD | 23.4±2.6 | 23.2±2.8 | 0.705 |  |  |
| **Initial presenting symptoms,** n% |  |  |  |  |  |
| jaundice | 12(46.2) | 26(54.2) | 0.510 |  |  |
| abdominal pain | 17(65.4) | 32(66.7) | 0.911 |  |  |
| **Type of surgery,** n% (n=73) |  |  |  |  |  |
| major hepatectomy | 21(80.8) | 35(74.5)* | 0.542 |  |  |
| major hepatectomy+PD | 5(19.2) | 12(25.5) |  |  |  |
| **CA19-9 level,** mean±SD | 517.8±667.0 | 594.5±679.3 | 0.640 |  |  |
| **CEA level,** mean±SD | 5.7±8.3 | 8.9±15.7 | 0.341 |  |  |
| **Total bilirubin levels,** mean±SD | 109.9±118.8 | 117.1±133.1 | 0.819 |  |  |
| **ALT levels,** mean±SD | 144.9±159.8 | 92.1±82.5 | 0.125 |  |  |
| **Preoperative biliary drainage,** n% | 10(38.5) | 22(45.8) | 0.541 |  |  |
| **Operation duration,** mean±SD | 337.8±80.3 | 340.3±73.9 | 0.936 |  |  |
| **Intraoperatve blood soss,** mean±SD | 530.8±342.7 | 488.5±319.6 | 0.591 |  |  |
| **Blood transfusion volume,** mean±SD | 1409.6±1867.2 | 1459.4±3091.3 | 0.941 |  |  |
| **Intraoperative hemorrhage,**n% | 0(0) | 4(8.3) | 0.291 |  |  |
| **Tumor size,** mean±SD | 7.02±1.97 | 7.49±2.84 | 0.461 |  |  |
| **Tumor location** |  |  |  |  |  |
| Bottom | 3(11.5) | 9(18.8) | 0.354 |  |  |
| Body | 4(15.4) | 9(18.8) | 0.618 |  |  |
| Neck | 13(50) | 15(31.3) | 0.112 |  |  |
| Uncertain | 6(23.1) | 15(31.3) | 0.356 |  |  |
| **Tumor differentiation** |  |  |  |  |  |
| Well | 1(3.8) | 4(8.3) | 0.651 |  |  |
| Moderately | 24(92.3) | 43(89.6) | 0.702 |  |  |
| Poorly | 1(3.8) | 1(2.1) | 1.00 |  |  |
| **Peripheral tissue invasion status** |  |  |  |  |  |
| perineural invasion | 11(42.3) | 25(52.1) | 0.422 |  |  |
| vascular invasion | 21(80.8) | 38(79.2) | 0.931 |  |  |
| common bile duct invasion | 6(23.1) | 5(10.4) | 0.144 |  |  |
| cancerization of duct | 1(3.8) | 3(6.3) | 0.756 |  |  |
| **Lymphatic metastasis status** |  |  |  |  |  |
| N0 | 17(65.4) | 30(62.5) | 0.806 |  |  |
| N1 | 7(26.9) | 18(37.5) | 0.358 |  |  |
| N2 | 2(7.7) | 0 | 0.12 |  |  |
| **GBC staging** |  |  |  |  |  |
| IV A | 24(92.3) | 48(100) | 0.12 |  |  |
| IV B | 2(7.7) | 0(0) |  |  |  |
| **Types of histology** |  |  |  |  |  |
| adenocarcinoma | 22(84.6) | 45(93.8) | **0.048** | 0.39(0.11-1.59) | 0.192 |
| squamous-cell carcinoma | 2(7.7) | 2(4.2) | 0.522 |  |  |
| adenosquamous carcinoma | 1(3.8) | 1(2.1) | 0.655 |  |  |
| neuroendocrine tumor | 1(3.8) | 0(0) | 0.351 |  |  |
| **Major postoperative complications** | 15 | 20.3 |  |  |  |
| infection | 2(7.7) | 4(8.3) | 0.923 |  |  |
| delayed gastric emptying | 1(3.8) | 3(6.3) | 0.662 |  |  |
| biliary fistula | 0(0) | 1(2.1) | 1.00 |  |  |
| others | 1(3.8) | 3(6.3) | 0.662 |  |  |
| **Recurrence sites** (n=61) |  |  |  |  |  |
| locally | 16(76.2) | 17(42.5) | **0.012** | 1.68(1.20-1.99) | 0.016 |
| distant | 5(23.8) | 23(57.5) |  |  |  |
| **Adjuvant chemotherapy cycles,** mean±SD | 8.20±2.52 | 4.50±3.50 | **0.016** | 1.36(1.09-1.86) | 0.026 |

SD: Standard deviation; GBC: Gallbladder cancer; IQR: interquartile range; PD: pancreatoduodenectomy; BMI: body mass index; CEA: carcinoembryonic antigen; ALT: alaninetransaminase;
